# Acquired colonic atresia in children: a report of three cases and review of the literature

**DOI:** 10.1093/jscr/rjae012

**Published:** 2024-08-23

**Authors:** Dina Fouad, George S Bethell, Nigel J Hall

**Affiliations:** Department of Paediatric Surgery and Urology, Southampton Children’s Hospital, Southampton SO16 6YD, United Kingdom; Department of Paediatric Surgery and Urology, Southampton Children’s Hospital, Southampton SO16 6YD, United Kingdom; University Surgery Unit, Faculty of Medicine, University of Southampton, Southampton SO16 6YD, United Kingdom; Department of Paediatric Surgery and Urology, Southampton Children’s Hospital, Southampton SO16 6YD, United Kingdom; University Surgery Unit, Faculty of Medicine, University of Southampton, Southampton SO16 6YD, United Kingdom

**Keywords:** colonic atresia, cardiovascular disease, infant, intestinal obstruction

## Abstract

We describe cases of three infants who developed acquired colonic atresia presumed secondary to significant systemic cardiovascular compromise and in the absence of necrotizing enterocolitis. An acquired colonic atresia may present as feed intolerance and should be investigated with a lower gastrointestinal contrast study. We would also recommend routine lower gastrointestinal contrast study prior to stoma closure in an infant with history of significant cardiovascular compromise, even in the absence of significant widespread colonic inflammation such as necrotizing enterocolitis.

## Introduction

Intestinal strictures in children are a well recognized sequelae of a number of inflammatory conditions such as necrotizing enterocolitis (NEC) or Crohn's disease. Less frequently, complete occlusion of the intestine may occur in response to an inflammatory insult or due to an obstructive pathology such as adhesive bowel obstruction or intusussception, thus resulting in an acquired intestinal atresia. Where such acquired atresias occur, they are most commonly found in small bowel rather than colon [1–5]. We report three unusual cases of acquired colonic atresia in infants, all of whom had underlying cardiovascular compromise for different reasons without NEC. We also provide a review of the existing literature on this topic. We believe this is the first series to describe acquired colonic atresia in infants with the suspected aetiology being intestinal vascular insufficiency and not secondary to NEC.

## Materials and methods

For the three cases we performed a retrospective casenote review, extracting all relevant clinical details. We conducted a literature search using PubMed, Cochrane, OVID Medline, and EMBASE using keywords ‘acquired atresia’ and ‘colon’. Variations of these words were also searched. All articles available in english and describing children or infants were reviewed.

## Case series

### Case 1

An ex 36 week male infant (birthweight 2.5 kg) with an antenatal diagnosis of atrial flutter and a single umbilical artery. Supraventricular tachycardia (SVT) with poor perfusion was diagnosed at birth, prompting treatment with adenosine, amiodarone and subsequently propranolol. Due to other dysmorphic features, a diagnosis of Kleefstra syndrome was confirmed prior to discharge home with no gastrointestinal concerns.

**Figure 1:**
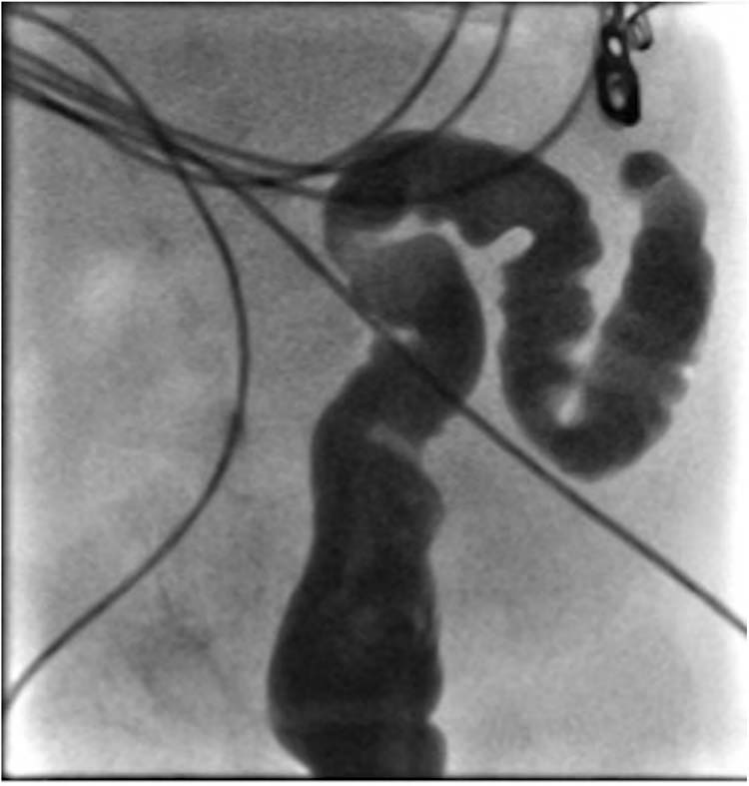
Contrast enema showing failure of passage of contrast beyond descending colon.

**Figure 2:**
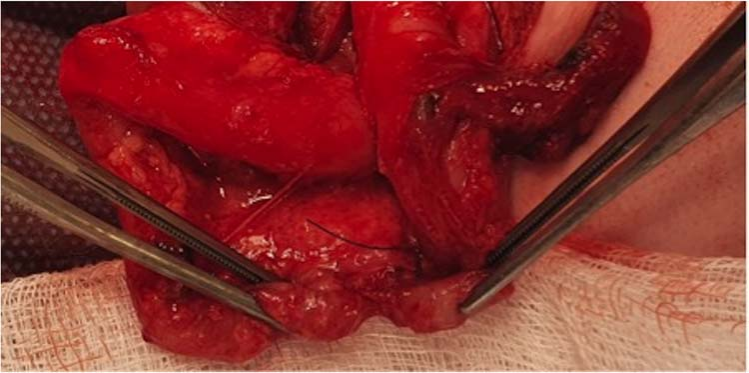
Intra-operative photograph of acquired colonic atresia in descending colon consistent with radiological findings in Fig. 1.

He presented at 6 weeks of age with circulatory shock requiring inotropic support and a short history of sudden abdominal distension and non bilious vomiting. He had been having regular episodes of SVT, all self limiting. Laparotomy demonstrated malrotation and a 540°midgut volvulus with extensive bowel necrosis. A fulcrum of ileum was densely adherent to the descending colon. Following intestinal resection he was left with 40 cm of small bowel from the duodenojejunal flexure, 2 cm of distal ileum and an intact colon. Following recovery from this acute episode he was managed for intestinal failure. A contrast enema was performed 10 days later in advance of stoma closure to examine the area of descending colon to which the ileum had been adherent at laparotomy. This demonstrated failure of passage of contrast proximal to the mid descending colon consistent with acquired colonic atresia (Fig. 1). Due to ongoing high stoma output and inability to progress enteral feeds, the infant proceeded to laparotomy, closure of jejunostomy, resection of colonic atresia, and anastomosis at 3 months of age. Macroscopic evidence of the atresia was encountered (Fig. 2). Histology reported a narrow calibre colon to either side of the atresia with fibrotic submucosa, focal fibrovascular proliferation, and clusters of giant cells. He subsequently achieved enteral autonomy.

### Case 2

A 5-week-old male infant with trisomy 21 and a small ventricle septal defect (VSD), who required intensive care admission for respiratory syncytial virus (RSV) bronchiolitis complicated by sepsis. He required significant fluid resuscitation and inotropic support for significant hypotension for 72 hours. Following this, he developed abdominal distension, rectal bleeding, and bilious gastric aspirates. An abdominal radiograph showed evidence of bowel wall thickening and linear pneumatosis intestinalis. A diagnosis of ischemic colitis was suspected and treatment with bowel rest and broad-spectrum intravenous antibiotic therapy was commenced. Following an initial improvement, he subsequently developed pneumoperitoneum and proceeded to laparotomy during which a caecal perforation was identified. A right hemicolectomy was performed with ileostomy and mucous fistula formation. The residual ileum and colon were macroscopically normal. Subsequent rectal biopsy excluded Hirschsprung’s disease. The baby recovered well and was discharged.

**Figure 3:**
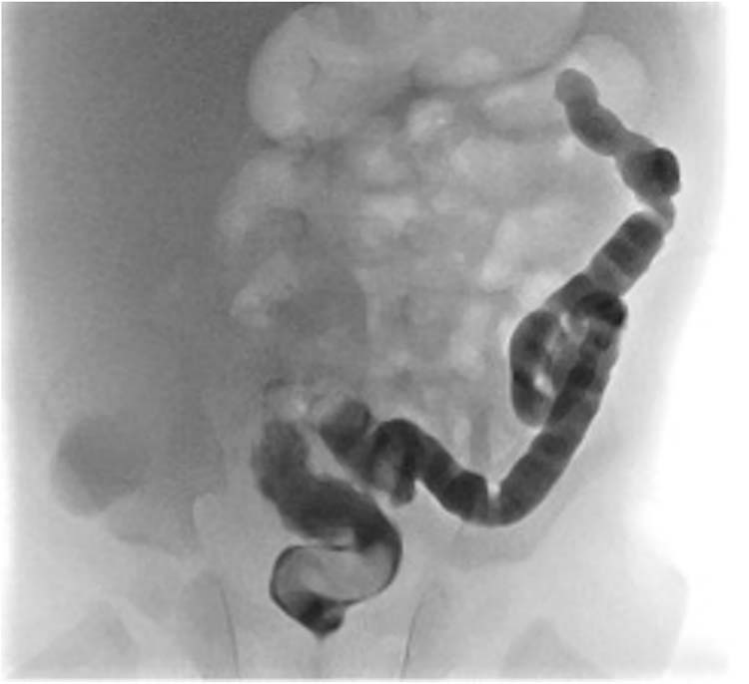
Contrast enema for case 2 showing complete failure of passage of contrast into distal transverse colon.

At elective ileostomy closure 2 months later, a colonic stricture was found 5 cm distal to mucous fistula, which was resected. Saline was instilled distal to the excised colonic stricture, which did not progress suggesting further distal obstruction. The decision was made to defer stoma closure at this stage. Subsequent colonoscopy revealed normal distal colon up to the level of the distal transverse colon at which point the colon was completely occluded in a blind ending pouch and on probing with a guide wire no lumen was found. Atresia was confirmed with contrast enema (Fig. 3). At 8 months of age, he underwent laparotomy and resection of a 10 cm section of transverse colonic atresia. Histology reported narrowing of the transverse colon, focal granulation tissue, and mucosal web formation. His recovery and subsequent follow-up have been uneventful.

### Case 3

**Figure 4:**
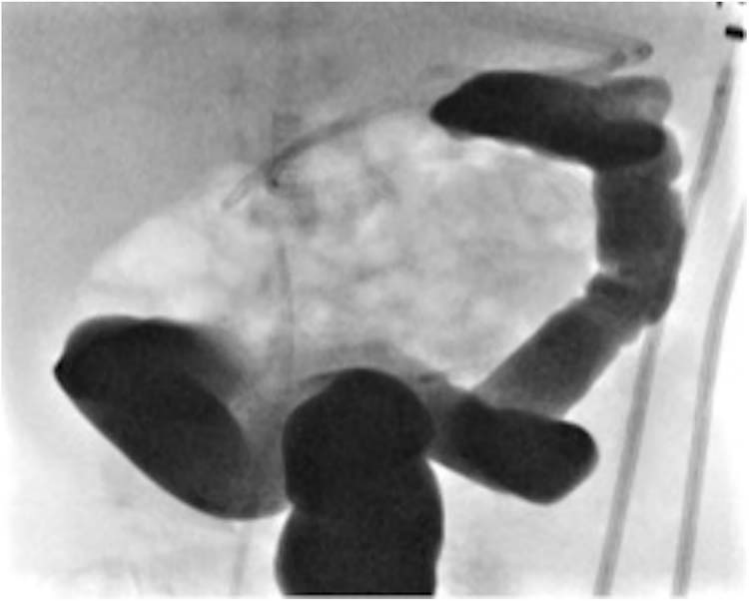
Contrast enema for case 3 showing complete failure of passage of contrast into distal transverse colon.

A female infant born at 37 weeks (birth weight 1.9 kg) and a neonatal course complicated by significant respiratory distress and bilateral pneumothoraces, also found to have a hypoplastic aortic arch on echocardiogram. On day 4 of life, she underwent an aortic arch repair (bypass time - 2 h 44 min, clamp time 24 min) and the chest was left open. Inotropes were required post operatively (3 days of vasopressin, 4 days of dopamine, and 19 days of milrinone). At 4 weeks of age, she underwent chest closure. At 5 weeks of age, she developed feed intolerance, abdominal distension, and bilious nasogastric aspirates. She was treated with bowel rest and intravenous antibiotics for 1 week. At 9 weeks of age, she was investigated for ongoing inability to advance feed and found to have distal transverse colonic obstruction (Fig. 4). At laparoscopy, a dilated proximal transverse colon was encountered with a clear transition point to collapsed distal colon. However, the serosal surface was intact and uninterrupted. An enterotomy made at the transition point revealing complete occlusion of the lumen by a mucosal web consistent with an acquired colonic atresia. A transverse loop colostomy was fashioned at this point and the baby recovered well. She is awaiting colostomy closure. A diagnosis of Turner’s syndrome was later confirmed. We have included a summary of cases 1, 2, and 3 in Table 1.

**Table 1:** Summary of cases.

	**Age at presentation**	**Co-morbidities**	**Cardiovascular events precipitating acquired atresia**	**Site of atresia**
Case 1	43 days	SVT	Presented shocked due to malrotation with volvulus requiring multiple inotropes	Descending colon
Case 2	37 days	Trisomy 21 and small VSD	Presented shocked post RSV bronchiolitis. Prolonged inotropic requirement	Distal transverse colon
Case 3	37 days	Hypoplastic aortic arch	Cardiopulmonary bypass Prolonged inotropic requirement post operatively.	Distal transverse colon

**Table 2:** Summary of nine previous cases of acquired colonic atresia.

**Author**	**Year**	**Case summary**	***n***
Guttman [5]	1976	5 week old with colonic atresia post NEC	1
Beardmore [2]	1978	8 week and 11 week olds with colonic atresia post NEC	2
Mares [1]	1993	4 cases of colonic atresia post NEC	4
Rattan [8]	2017	4 month old with colonic atresia and unknown aetiology	1
Parida [9]	2022	6 week old with colonic atresia post NEC	1
**Total**			9

*n* = number of patients.

## Discussion

We report three cases of acquired colonic atresia, all in infants with no pre-existing gastrointestinal symptoms two of whom had previously tolerated full enteral feeds and all of whom had previously had a normal stooling pattern with changing stool. We suspect a common aetiology for these cases since all three infants experienced significant cardiovascular compromise, which likely contributed to poor bowel perfusion and ischaemia followed by development of an acquired atresia. This is comparable with the ‘ischaemic hit theory’ for neonates born with an intestinal atresia. Although acquired atresia has been previously reported, this typically involves the small bowel whereas these three cases involved the colon. We postulate that the colon is more sensitive to intestinal ischaemia than the small bowel in similarity to the observed phenomenon that infants with congenital cardiac disease who develop inflammation, ischaemia, or necrosis of the intestine often described as ‘cardiac NEC’, affects primarily the colon as opposed to the small bowel [6]. It is unclear whether this is due to selective mesenteric blood flow redistribution or simply the well-recognized water-shed area in the colon given that this area is the site of atresia in all cases within this current series.

Of note all three infants in this series had a history consistent with acute and/or chronic cardiovascular insufficiency including recurrent SVT and an episode of circulatory collapse in case 1, circulatory collapse requiring inotropes during septic shock with a history of a VSD case 2, and post cardiopulmonary bypass for a hypoplastic aortic arch repair with post-procedural inotrope requirement case 3. This supports the vascular insult theory as the aetiology of ischaemia leading to acquired atresia. We hypothesize that the ischaemic component here may predispose to complete atresia of the colon as opposed to stricturing, more commonly seen following an inflammatory process such as NEC or Crohn’s disease [7]. A further interesting observation in two of these cases is that the atresia appeared to be caused by a mucosal web without obvious interruption of the serosa.

Our literature review identified just nine previous cases of acquired colonic atresia in infants and children [1, 2, 5, 8, 9]. All of these cases had a precipitating history of NEC except from one where the cause was unclear and there was no pre-existing cardiovascular disease, which was seen in our cases. These cases are summarized in Table 2.

In summary, we describe three infants who developed acquired colonic atresia presumed secondary to significant systemic cardiovascular compromise without NEC. An acquired colonic atresia may present as feed intolerance and should be investigated with a lower gastrointestinal contrast study. Lower gastrointestinal contrast studies are routinely undertaken prior to stoma closure following NEC [10]; however, we would recommend this investigation prior to stoma closure in any infant with history of significant cardiovascular compromise.
